# 1123. Appropriateness of Antibiotic Prescribing Through the COVID-19 Pandemic and Associated Telehealth Visits

**DOI:** 10.1093/ofid/ofab466.1316

**Published:** 2021-12-04

**Authors:** Bethany A Wattles, James A Stahl, Kahir S Jawad, Yana Feygin, Maiying Kong, Navjyot Vidwan, Michelle D Stevenson, Michael J Smith

**Affiliations:** 1 University of Louisville School of Medicine, Lexington, Kentucky; 2 Norton Children’s Hospital, Louisville, Kentucky; 3 University of Louisville, Louisville, Kentucky; 4 Norton Children’s Medical Group and University of Louisville, Louisville, Kentucky; 5 Duke University Medical Center, Durham, North Carolina

## Abstract

**Background:**

The COVID-19 pandemic and resulting mitigation strategies have impacted rates of outpatient infections and delivery of care to pediatric patients. Virtual healthcare was rapidly implemented but much is unknown about the quality of care provided in telehealth visits. We sought to describe changes in visits throughout the pandemic and evaluate the appropriateness of antibiotic prescribing.

**Methods:**

We utilized EHR data from a large health care system that provides primary care via pediatric, family medicine, and urgent care clinics. We included outpatient visits from 1/1/19 - 4/30/21 for children < 20 years. The COVID-19 era was defined as after March 2020. Visits were labeled as virtual according to coded encounter or visit type variables. The appropriateness of antibiotic prescriptions was assigned using a previously published ICD-10 classification scheme that defines each prescription as appropriate, potentially appropriate, or inappropriate (Chua, et al. BMJ, 2019).

**Results:**

There were 805,130 outpatient visits during the study period. The mean rate of antibiotic prescriptions in the pre-pandemic period was 23% (range 17-26% per month) and 11% (range 9-15%) in the COVID-19 era. Mean rates of inappropriate prescribing were 17% (range 14-20% per month) and 20% (range 19-22%), respectively (Figure 1). Coded virtual visits during the COVID-19 era were uncommon (1-2%) with the exception of April and May 2020 (11% and 5%, respectively). During the COVID-19 era, approximately 9% of telehealth visits resulted in antibiotics, compared to 11% of in-person visits (Table 1). Virtual visits had lower rates of inappropriate and appropriate prescribing, but higher rates of potentially appropriate prescribing (Table 1).

Visits and associated antibiotic prescribing in the pre-pandemic and COVID-19 era

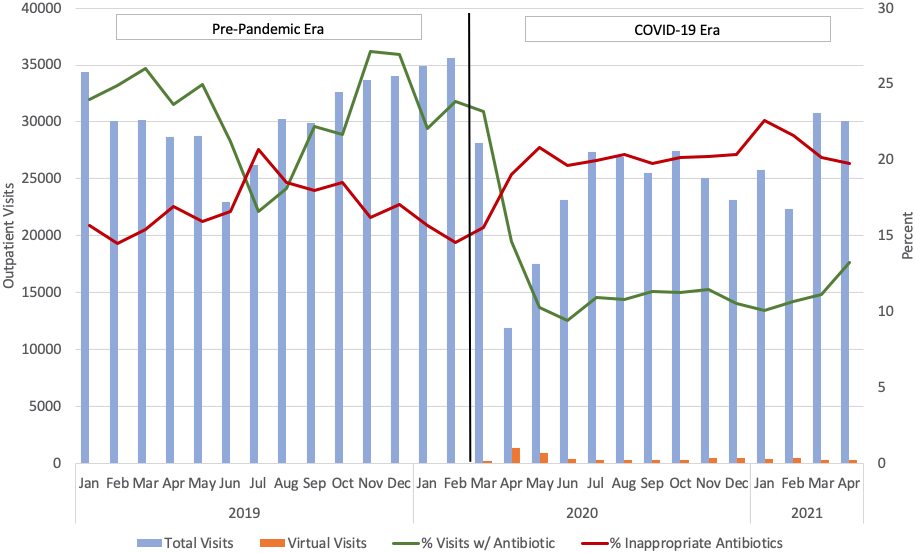

Appropriateness of antibiotic prescribing in the COVID-19 era, by visit type

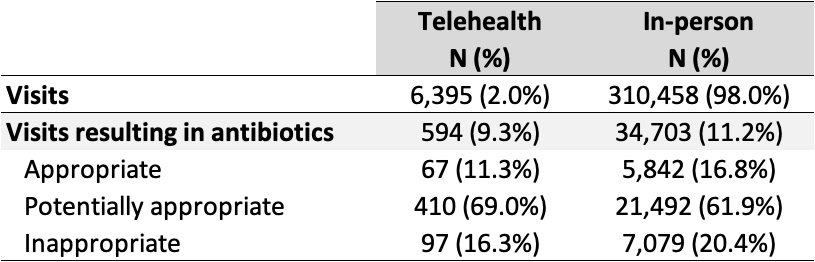

**Conclusion:**

Rates and volume of antibiotic prescribing in outpatient pediatric visits have declined in the COVID-19 era, while rates of inappropriate prescribing have increased slightly. Our study suggests use of telehealth for pediatric visits was minimal and led to higher prescribing rates for “potentially appropriate” indications. This could be explained by a lack of clinical certainty in conditions such as otitis media and pharyngitis in virtual visits.

**Disclosures:**

**Bethany A. Wattles, PharmD, MHA**, **Merck** (Grant/Research Support, Research Grant or Support) **Yana Feygin, Master of Science**, **Merck** (Grant/Research Support, Research Grant or Support) **Michelle D. Stevenson, MD, MS**, **Merck** (Grant/Research Support) **Michael J. Smith, MD, M.S.C.E**, **Merck** (Grant/Research Support)**Pfizer** (Grant/Research Support)

